# Investigating the role of the innate immune response in relapse or blast crisis in chronic myeloid leukemia

**DOI:** 10.1038/s41375-020-0771-7

**Published:** 2020-02-20

**Authors:** Weiqi Huang, Bin Liu, Elizabeth A. Eklund

**Affiliations:** 1 The Feinberg School, Northwestern University, Chicago, IL USA; 2 Jesse Brown Veterans Health Administration Medical Center, Chicago, IL USA

**Keywords:** Chronic myeloid leukaemia, Chronic myeloid leukaemia

## Abstract

Chronic myeloid leukemia (CML) is characterized by expression of the tyrosine kinase oncogene, Bcr–abl. Tyrosine kinase inhibitors (TKI) induce prolonged remission in CML, and therapy discontinuation is an accepted approach to patients with reduction in Bcr–abl transcripts of four logs or greater. Half such individuals sustain a therapy free remission, but molecular mechanisms predicting relapse are undefined. We found relative calpain inhibition in CML cells with stabilization of calpain substrates, including βcatenin and Xiap1. Since the Survivin gene is activated by βcatenin, this identified two apoptosis-resistance mechanisms. We found that Survivin impaired apoptosis in leukemia stem cells (LSCs) and Xiap1 in CML granulocytes. Consistent with this, we determined treatment with an inhibitor of Survivin, but not Xiap1, prevented relapse during TKI treatment and after therapy discontinuation in a murine CML model. By transcriptome profiling, we identified activation of innate immune response pathways in murine CML bone marrow progenitors. This was increased by TKI treatment alone, but normalized with addition of a Survivin inhibitor. We found that activation of the innate immune response induced rapid blast crisis in untreated CML mice, and chronic phase relapse during a TKI discontinuation attempt. These results suggest that extrinsic stress exerts adverse effects on CML-LSCs.

## Introduction

CML is characterized by translocation of chromosomes 9 and 22 with consequent expression of Bcr–abl; an oncogene tyrosine kinase [1]. Development of Bcr-abl-specific tyrosine kinase inhibitors (TKIs) improved outcomes in CML, and patients with optimal TKI responses may have a normal lifespan [2–4]. However, long term TKI-treatment is complicated by side effects and substantial costs to individuals and health care systems [4, 5]. In clinical trials, to investigate the feasibility of TKI-discontinuation, half of CML patients with at least a 3 log reduction in Bcr–abl transcripts (≥0.1% International Standard) sustained a therapy free remission (TFR) [6–9]. Nearly all relapsing subjects achieved a second molecular remission and current guidelines permit discontinuing therapy in select patients with ≥4 log transcript reduction (≥0.01% IS) [10]. However, molecular predictors of sustained TFR vs. relapse are undefined.

Relapse after therapy discontinuation requires that some LSCs persist during TKI-treatment. Since TKIs efficiently inhibit proliferating CML progenitors, persisting LSCs are hypothesized to be quiescent and apoptosis resistant [11]. We found relatively increased expression of Fap1 (Fas-associated phosphatase 1) in CML-LSCs, with consequent inhibition of Fas and Gsk3β [12–15]. This induced Fas-resistance and enhanced transcription of βcatenin target genes, including *BIRC5* (encoding Survivin). In a murine model of CML, we determine treatment with Imatinib (IM; a TKI inhibitor) plus a Fap1-blocking peptide prevented chronic phase (CP) relapse or blast crisis (BC) progression, although IM alone did not [12]. And, mice treated with IM plus Fap1-blocking peptide sustained remission after therapy discontinuation, although 60% of mice treated with IM alone relapsed.

We found expression endogenous calpain inhibitors (Gas2 and calpastatin) was also increased in human CD34^+^CML cells or Bcr-abl-transduced murine progenitors compared with control cells [16, 17]. This stabilized calpain substrates, including βcatenin and Xiap1 [17]. Consistent with this, Bcr–abl expression in bone marrow progenitors induced a calpastatin/calpain-dependent increase in Xiap1 in CML granulocytes, but a Gas2/calpain-dependent increase in βcatenin and Survivin [17]. In the current study, we investigate contributions of Survivin or Xiap1 to CML-LSC persistence and relapse. Unlike Fap1, translationally relevant inhibitors of these proteins are available [18, 19].

## Materials and methods

### Quantitative PCR

RNA was isolated with Triazol reagent. Primers were designed with Applied Biosystems software (Grand Island, NY), and PCR performed by SYBR green method. Result were normalized to 18S and actin. Four independent experiments were performed in triplicate.

### Flow cytometry

Cells were analyzed on a Becton-Dickinson FACScan (Cambridge, MA). For apoptosis, cells were incubated 12 h with IM (2 μM), Ym155 (10 nM), Embelin (10 μM) or buffer control; 24 h with Fas antibody (5 μg/ml CH11; BD Bioscience Inc., San Jose CA) or buffer control; labeled with PE-conjugated CD34 antibody, and analyzed by the Annexin V-Apoptosis Detection Kit I (eBioscience, San Diego CA). Four independent experiments were performed in duplicate. Variance within groups was not significantly different for various conditions.

### Murine bone marrow transduction and transplant

293T cells were transfected with p210-Bcr-abl-MiGR1 (from Dr Ravi Bhatia, University of Alabama, Birmingham) and pCL-Eco plasmids. This line was verified annually by STR and tested every 6 months for mycoplasma. Supernatants were collected after 48 h [12]. Primary bone marrow donors (C57/BL6 mice) were treated with 150 mg/kg of 5-flurouracil by intraperitoneal injection (IP) and bone marrow harvested after 4 days [12]. Cells were incubated with retroviral supernatant (~10^7^ pfu/ml) supplemented with polybrene (6 μg/ml) in DME, 10% FCS, 1% pen-strep, 10 ng/ml IL-3, 100 ng/ml Scf, 10 ng/ml IL-6 (R & D Systems Inc., Minneapolis, MN) [12, 20]. Transgene expression was confirmed by Bcr–abl PCR and GFP flow cytometry.

Lethally irradiated, syngeneic recipients were injected with 1 × 10^6^ transduced cells and sacrificed when peripheral WBC > 30,000 with >50% granulocytes but <5% blasts. Equal numbers of male and female mice were used. Bone marrow was transplanted into sublethally irradiated secondary recipients (2 × 10^6^ cells). Four weeks later, secondary recipients were IP injected with IM (100 mg/kg/day), Ym155 (5 mg/kg/day), Embelin (10 mg/kg/day), IM+Ym155 or Embelin, or saline (10/group). Each cohort included recipients from four different donors, and initial peripheral blood counts were not significantly different between groups. At 24 weeks, 2 × 10^6^ bone marrow cells from secondary recipients in molecular remission (≥3 log bone marrow Bcr–abl transcript reduction vs. untreated mice) were transplanted into sublethally-irradiated tertiary recipients. Tertiary recipients were observed without treatment. Ten mice were used per cohort for 80% power in a one sided test with continuous measurement (α = 0.05). This allows detection of differences between experimental groups occurring at a rate of 40%. All mice were included in the analysis and there was no preselection of groups. Variance within groups was not significantly different for the cohorts and was significantly different than variance between groups by ANOVA. Peripheral blood count data and survival were used to determine study results and no blinding was required.

Tail vein blood was obtained for automated counting. Blast counts were determined on May–Grunwald–Giemsa stained peripheral smears (300 cells/slide).

### Imatinib resistance assay

Transduced murine bone marrow was cultured in IM at an initial dose of 0.2 μg/ml; increasing to 2.0 μg/ml over 6 weeks (vs. sham) [12]. Ym155 or Embelin were added to some cultures and cells counted weekly. Two independent experiments were performed in triplicate.

### Emergency granulopoiesis

Mice were injected IP with ovalbumin/aluminum chloride (i.e., Alum) or saline every 4 weeks starting 4 weeks after secondary or tertiary transplantation with Bcr–abl^+^ bone marrow (8 mice/group), as described [21–23]. Peripheral blood counts were determined every 2 weeks. Secondary recipients received 5 × 10^5^ bone marrow cells from primary recipients. Tertiary recipients received 5 × 10^5^ bone marrow cells from secondary recipients in IM-induced molecular remission at 24 weeks.

### RNA sequencing and gene ontology

Stranded mRNA-Seq was conducted in the Northwestern University NUSeq Core with RNA from GFP ^+^ Lin^−^ murine bone marrow cells (4/cohort, non-pooled samples). RNA quality was determined using an Agilent Bioanalyzer 2100 (Agilent Research Laboratories, Santa Clara, CA). Libraries were prepared with the TruSeq Stranded mRNA kit (Illumina Inc, San Diego, CA) and validated. Single-end, 75 bp reads were generated using an lllumina NextSeq 500 Sequencer. DNA read quality was evaluated using FastQC. Adapters were trimmed and reads of poor quality or aligning to rRNA sequences filtered. Cleaned reads were aligned to the *Mus musculus* genome using STAR and read counts calculated by htseq-count in conjunction with mm10 gene annotation file (http://genome.ucsc.edu). Differential expression was determined using DESeq2 [24]. Statistical significance of differentially expressed genes was an FDR-adjusted *p* value < 0.05.

### Statistical analysis

Significance was determined by a two-tailed Student’s *t* test or ANOVA using SigmaStat (Systat Software Inc, San Jose CA). Data is reported as average ± SD with *p* ≤ 0.02 considered significant. Survival/relapse rate differences were analyzed by the Mann–Whitney Rank Sum test. Blood counts in treatment cohorts were analyzed by the Kruskal–Wallis One Way Analysis of Variance on Ranks.

### Human and murine studies

Approved by Institutional Review Board or Animal Care and Use Committee of Northwestern University and Jesse Brown VA. Informed consent was obtained from all subjects.

## Results

### Survivin and Xiap1 influenced apoptosis in CML

We first investigated the impact of translationally relevant inhibitors of Survivin or Xiap1 on CML cells in vitro. We found increased βcatenin protein and Survivin mRNA in human CD34^+^CML cells compared with controls, but no increase in Xiap1 (Supplementary Fig. 1A, B). Conversely, Xiap1, but not βcatenin or Survivin, increased after ex vivo differentiation of CD34^+^CML cells with G-CSF; similar to our studies with Bcr-abl-transduced murine bone marrow [17]. In human or murine CML cells, ex vivo treatment with Ym155 decreased Survivin expression, and with Embelin decreased Xiap1, in a dose dependent manner (Supplementary Fig. 1C). Ym155 impairs Survivin expression by binding the *BIRC5* promoter, and Embelin enhances Xiap1 degradation by inhibiting caspase9 interaction [18, 19].

**Fig. 1:**
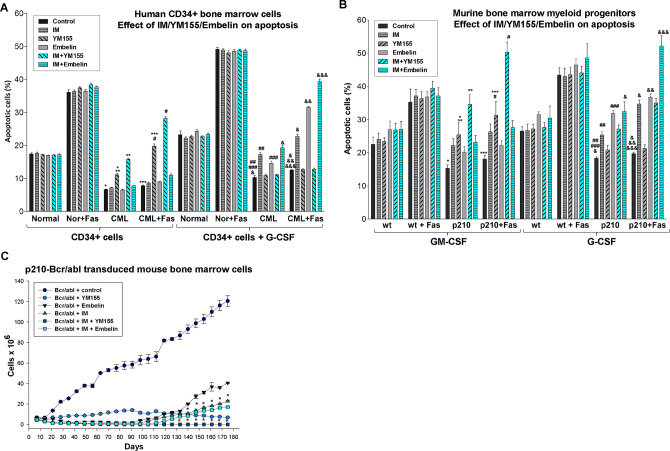
Differential activities of Survivin and Xiap1 in CML. **a** Ym155 enhanced apoptosis of human CD34^+^CML cells, but Embelin enhanced apoptosis during G-CSF-differentiation. Bone marrow CD34^+^cells were isolated from CP-CML or control subjects. Some cells were differentiated with G-CSF and/or treated with Imatinib (IM) ± inhibitors of Survivin (Ym155) or Xiap1 (Embelin). Cells were analyzed by flow cytometry for Annexin V. Significant differences indicated by *, **, ***, #, ##, ###, &, &&, or &&&. **b** Ym155 enhanced apoptosis in Bcr-abl-transduced murine bone marrow progenitors, but Embelin enhanced apoptosis during differentiation. Cells transduced with Bcr–abl or control vector were analyzed, as above. Significant differences indicated by *, **, ***, #, ##, ###, &, &&, or &&&. **c** Ym155 prevented in vivo emergence of IM resistance in Bcr-abl-transduced murine bone marrow progenitors. Cells were cultured in increasing IM ± Ym155 or Embelin. Significant differences indicated by *. For all comparisons *p* < 0.01, *n* = 6.

We found relative inhibition of Fas-induced and intrinsic apoptosis in human CD34^+^CML cells, with or without G-CSF differentiation (*p* < 0.01, *n* = 6 compared with controls) (Fig. 1a). Ym155 increased intrinsic and Fas-induced apoptosis of CD34^+^, but not G-CSF differentiated, CML cells. Adding IM to Ym155 enhanced Fas-responsiveness and intrinsic apoptosis in CD34^+^CML cells (*p* < 0.01, *n* = 6 with vs. without IM). IM or Embelin enhanced Fas-induced and intrinsic apoptosis in G-CSF-differentiated, but not CD34^+^, CML cells. Adding IM to Embelin enhanced Fas-induced apoptosis and normalized intrinsic apoptosis in differentiating CML cells (*p* < 0.001, *n* = 6 with vs. without IM). Apoptosis in control cells was not altered by any of these agents, with or without G-CSF.

We performed similar studies with murine bone marrow progenitor cells transduced with Bcr–abl or control vector (Fig. 1b). Similar to human cells, Bcr–abl decreased Fas-induced and intrinsic apoptosis, with or without G-CSF (*p* < 0.001, *n* = 6 compared with control). Effects of IM, Ym155, or Embelin on Bcr-abl-transduce murine cell populations were similar to effects on human CML samples. Therefore, we investigated the impact of inhibiting Survivin or Xiap1 on CML-LSC persistence during in vitro IM-treatment [12]. For these studies, Bcr-abl-transduced murine progenitor cells were cultured in a slowly increasing IM dose with or without Ym155, Embelin, or saline (Fig. 1c). IM, Embelin, or IM + Embelin initially suppressed growth, but cell numbers increased with time. Ym155 sustained growth suppression, but combination with IM resulted in total cell death by ~100 days.

### Survivin inhibition prevented TKI resistance and blast crisis in mice with CML

**Fig. 2:**
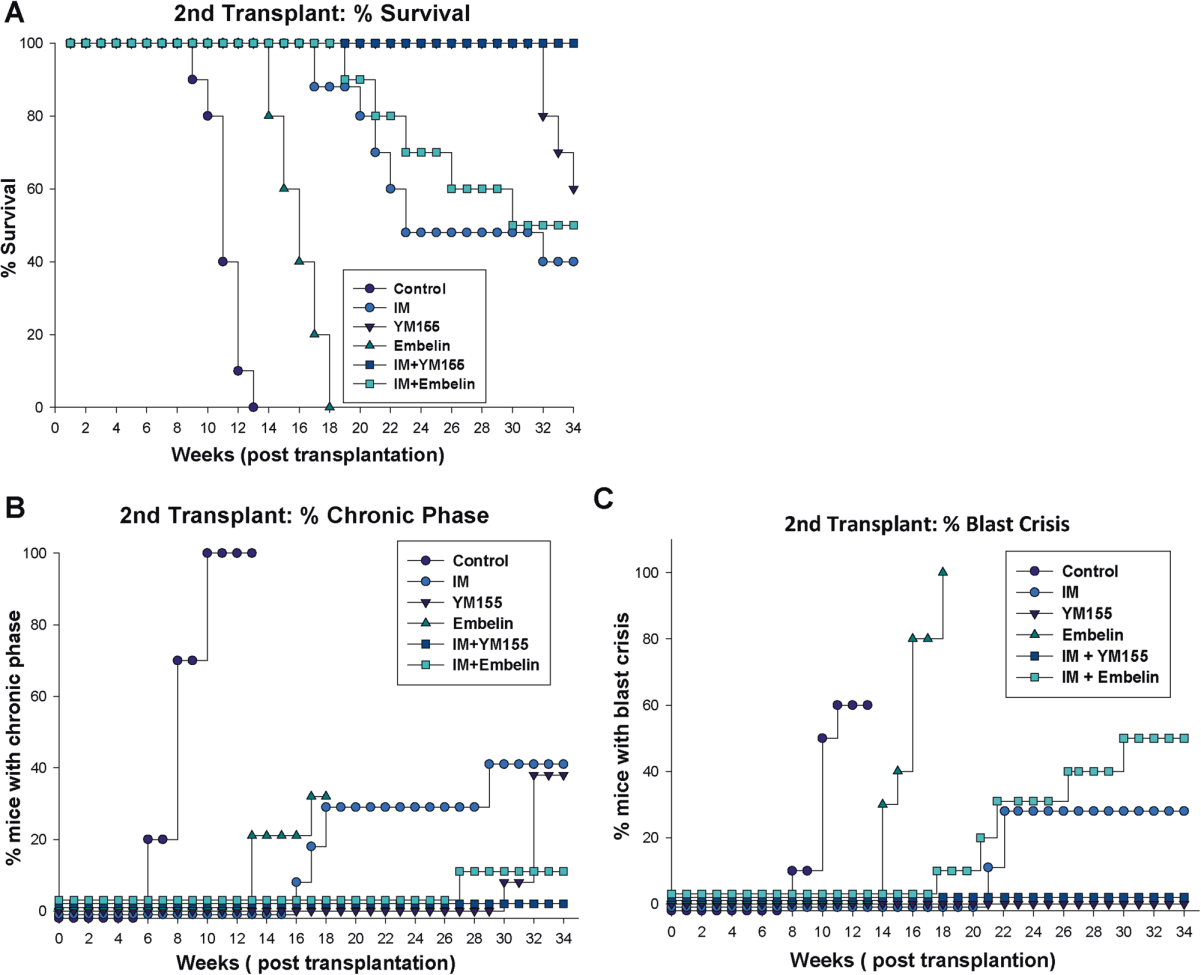
Ym155 prevented IM-resistance and BC in a murine CML model. Mice transplanted with Bcr-abl-transduced bone marrow were observed until CP. Secondary recipients of bone marrow from these mice were treated with IM ± Ym155 or Embelin. **a** Ym155 prolonged survival in IM treated mice, but Embelin did not. **b** Mice treated with IM or Ym155 relapsed in CP, but mice treated with IM + Ym155 or Embelin ± IM did not. **c** Treatment with Ym155 ± IM prevented BC. Embelin-treated mice relapsed in BC, and increased BC in IM treated mice.

We used an in vivo model to investigate the impact of Survivin or Xiap1 on CML [12]. For these studies, mice were transplanted with Bcr-abl-transduced bone marrow and observed until CP-CML developed. Bone marrow from these mice was transplanted into secondary recipients to generate cohorts with established CP-CML. Untreated secondary recipients died by 12–14 weeks (Fig. 2a); 40% with overwhelming CP (Fig. 2b) and the remainder progressing from CP to BC (Fig. 2c). In contrast, 50% of IM treated secondary recipients were alive at 24 weeks (*p* < 0.001, *n* = 10) and ~40% survived 30 weeks (human equivalent >40 years). All IM-treated mice achieved remission, but ~40% relapsed in CP, with half subsequently progressing to BC. All mice treated with Embelin alone developed BC by 18 weeks. Adding Embelin to IM did not prolong survival (*p* = 0.4, *n* = 10) and favored direct BC relapse. Survival was prolonged by Ym155 compared with untreated mice (*p* < 0.01, *n* = 10), and relapse was exclusively in CP. No mice treated with IM plus Ym155 relapsed during 35+ weeks.

Survivin expression in GFP^+^Lin^−^ bone marrow cells from secondary recipients was decreased by IM-treatment (*p* < 0.001, *n* = 3) (Supplemental Fig. 2A). Survivin increased during CP relapse and was greatest in BC; paralleling βcatenin (Supplemental Fig. 2B). Musashi-2 (Msi2) increases during BC and inhibits Apc translation; potentially stabilizing βcatenin [25, 26]. However, Msi2 was less in GFP^+^Lin^−^ cells from untreated secondary recipients compared with Lin^−^ control cells, increased with IM, and decreased upon CP relapse (Supplemental Fig. 2A). Apc protein abundance was similar in Lin^−^ control cells or GFP^+^Lin^−^ cells from untreated or IM-treated secondary recipients, but decreased in CP or BC (*p* ≤ 0.01, *n* = 3) (Supplemental Fig. 2B). Gli1 activates the survivin promoter and increases in BC [27, 28]. Gli1 expression also paralleled survivin in GFP^+^Lin^−^ cells from secondary recipients (Supplemental Fig. 2A).

### Survivin inhibition prevented relapse post TKI discontinuation in mice with CML

**Fig. 3:**
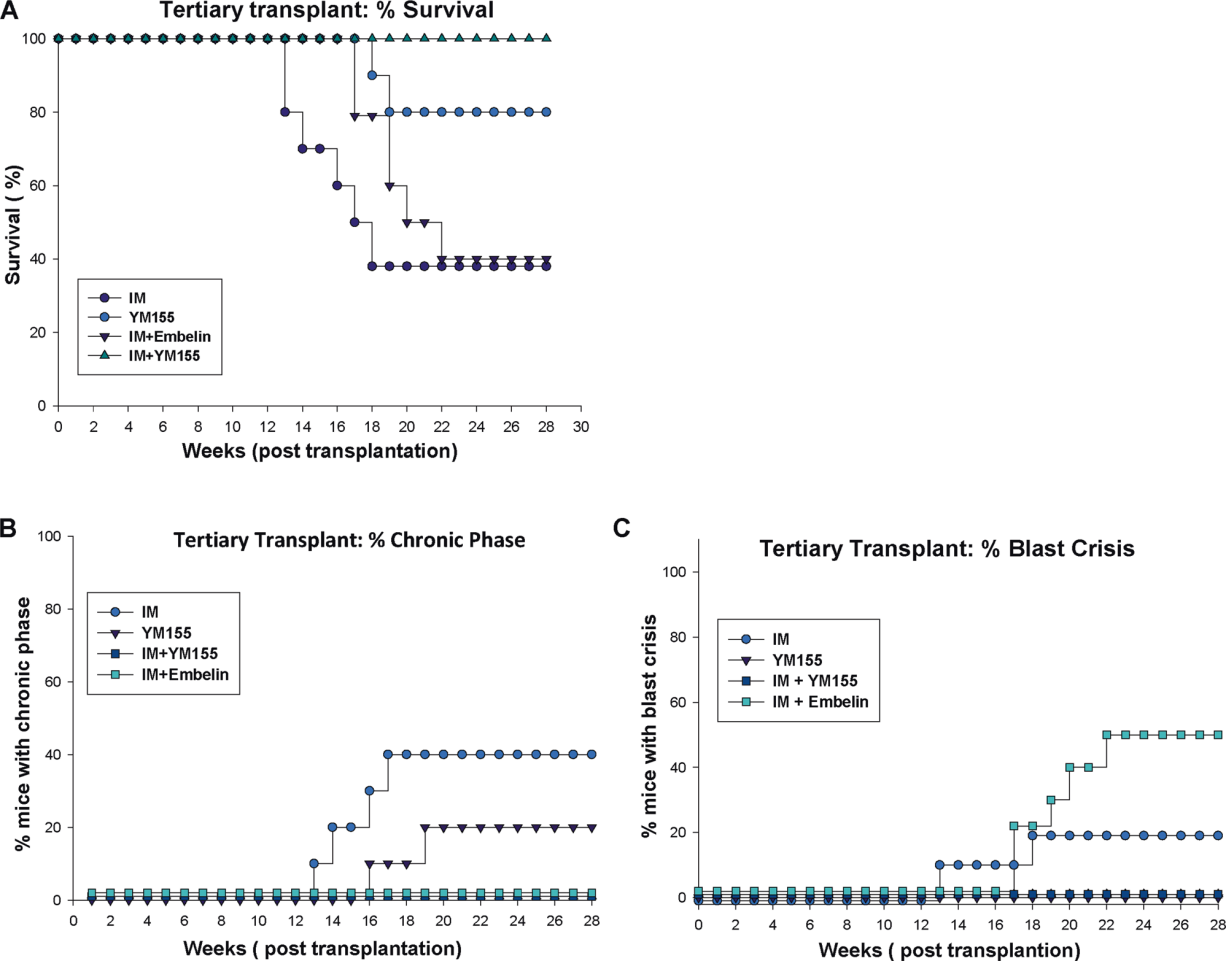
Addition of Ym155 to IM prevented relapse after therapy discontinuation in a murine CML model. Tertiary transplants were performed with bone marrow from secondary recipients of CP-CML bone marrow in molecular remission after 24 weeks of treatment with IM, Ym155, or IM + Ym155 or Embelin. Tertiary recipients were observed without treatment. **a** Tertiary recipients from Ym155-treated donors survived significantly longer than recipients from IM treated donors. All recipients from IM + Ym155 treated donors survived after therapy discontinuation. **b** No recipients from IM + Ym155-treated donors relapsed post therapy discontinuation, but 60% of recipients from IM-treated donors relapsed in CP. **c** BC increased significantly in recipients from donors treated with IM + Embelin vs. IM alone. No recipients from Ym155 ± IM-treated donors developed BC.

We used a tertiary transplant model to study relapse after therapy discontinuation [12]. In these studies, tertiary recipients of bone marrow from mice treated with IM, Ym155, IM+Ym155, or IM+Embelin were followed without additional treatment. Bone marrow donors were in molecular remission at 24 weeks (≥3.0 log decrease in Bcr–abl transcripts vs. untreated). Pre-discontinuation treatment with Ym155 alone significantly improved survival in tertiary recipients compared with IM alone (*p* < 0.001, *n* = 6) (Fig. 3a). Forty-five percent of recipients from IM-treated mice relapsed in CP by 18 weeks (Fig. 3b), with half progressing to BC (Fig. 3c). Only 20% of recipients from Ym155 treated mice relapsed in CP over 30 weeks (*p* < 0.001, *n* = 6), with half progressing to BC. However, all recipients of bone marrow from IM + Ym155 treated mice survived 35+ weeks without relapse. Survival in tertiary recipients was slightly prolonged by addition of Embelin to IM in secondary donors, but 70% of these mice had direct BC-relapse.

The same number of GFP^+^ cells were transplanted into all tertiary recipients, but addition of Ym155 to IM reduced Bcr–abl transcripts by 1.5 log vs. IM alone. Adding Ym155 to IM reduced the relative abundance of GFP^+^Lin^−^Sca1^+^ckit^+^ cells (89% for IM vs. 11% for IM+Ym155), but increased the abundance of GFP^+^Lin^+^Gr1^+^ cells (15% for IM vs. 86% for IM + Ym155; *n* = 4) (*p* < 0.001 for both). Bcr–abl copies/ GFP^+^Lin^+^ or GFP^+^Lin^−^ cell were similar in the two treatment groups. However, transcripts were greater in GFP^+^Lin^−^ vs. GFP^−^Lin^+^ cells (Lin^+^ = 28.1 ± 5.0 for IM and 22.3 ± 2.2 for IM + Ym155; Lin^−^ = 430 ± 10.5 for IM and 305 ± 10.0 for IM + Ym155) (*p* < 0.001, *n* = 4).

### Survivin inhibition during TKI treatment normalized innate immune response pathways

**Fig. 4:**
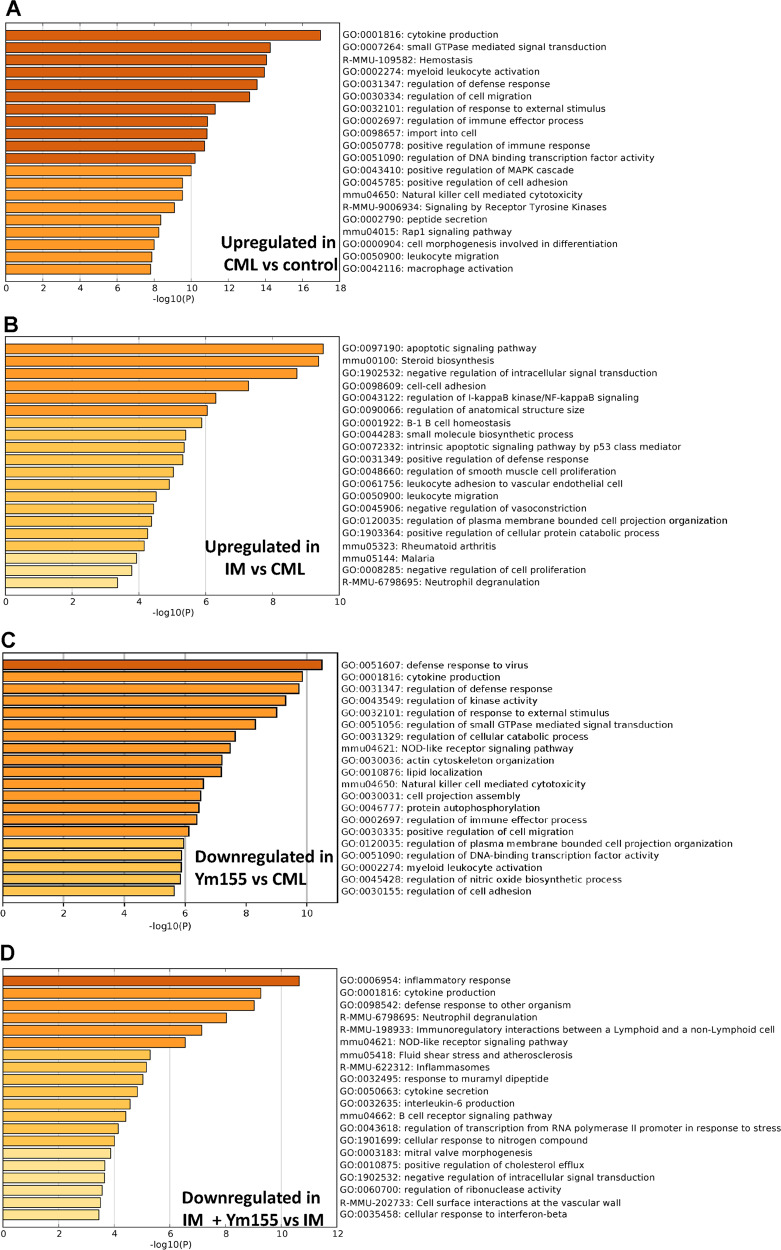
Innate immune response pathways were activated in the bone marrow of CML mice in IM-induced remission compared with IM + Ym155-induced remission. Secondary recipients of Bcr-abl-transduced bone marrow were treated with IM, Ym155, both or saline. GFP^+^Lin^−^ bone marrow cells from mice in molecular remission were analyzed by RNA sequencing. **a** Immune response pathways were increased in mice with untreated CP-CML compared with Lin^−^ cells from control mice. **b** Activity of pro-apoptotic and immune response pathways increased in IM-treated mice in remission compared with untreated CML. **c** Ym155-treatment decreased activity of immune response and kinase pathways compared with untreated CML mice. **d** Addition of Ym155 to IM decreased innate immune response pathway activity compared with IM alone.

To investigate mechanisms for LSC persistence and relapse post therapy discontinuation, we performed transcriptome analysis of GFP^+^Lin^−^ bone marrow cells. In mice with untreated CML (secondary recipients), we found increased activity of pathways involved in cytokine production and tyrosine kinase signaling compared with Lin^−^ cells from control mice (Fig. 4a). We also found relatively increased activity of pathways regulating the innate immune response, immune effector processes, and the defense response in CML. Expression of the leukemia suppressor Irf8/Icsbp was decreased and Gas2 increased in GFP^+^Lin^−^ vs. control Lin^−^ cells, consistent with prior work [29–31].

In GFP^+^Lin^−^ cells from mice with IM-induced molecular remission, activity of pathways involved in apoptosis, cellular catabolism, and negative regulation of proliferation were increased compared with untreated CML (Fig. 4b). However, activity of immune response pathways further increased with IM treatment, and Irf8/Icsbp and Gas2 expression was unaltered. In contrast, Ym155 increased Irf8/Icsbp expression, decreased Gas2, and decreased activity of defense response and immune effector pathways with or without IM (Fig. 4c, d). We therefore, investigated the functional significance of the innate immune response for LSC expansion and relapse.

### Emergency granulopoiesis enhanced blast crisis in untreated CML

We hypothesized that abnormalities in CML, such as decreased Icsbp/Irf8 expression, might prevent termination of a physiologically induced innate immune response; leading to CML progression. To explore this, we studied emergency (stress) granulopoiesis in mice with CP-CML. We previously found steady state failed to resume after stimulation of an emergency granulopoiesis response in *Irf8*^−/−^ mice and BC was accelerated [21]. Emergency granulopoiesis is an episodic process for granulocyte production in response to infectious challenge and a key component of innate immunity [32, 33]. It is studied in mice by injection of pathogens or an antigen/adjuvant combination (such as Alum) [21–23]. Alum induces the same cytokine and cellular response as pathogens, but without death or chronic infection of the mice.

**Fig. 5:**
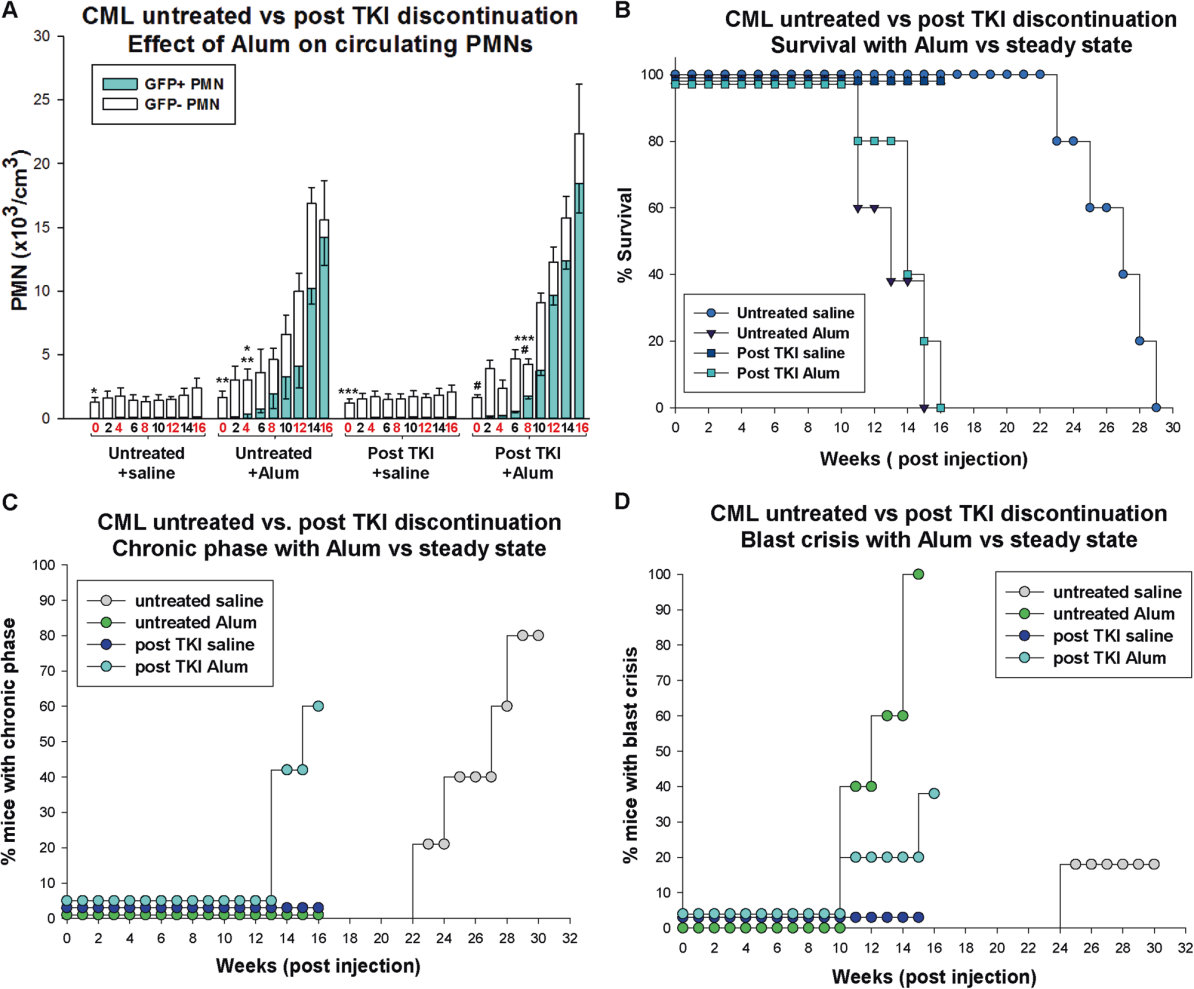
Emergency granulopoiesis influenced leukemogenesis in CML mice. Some secondary recipients of CP-CML bone marrow were injected with Alum or saline every 4 weeks. Other secondary recipients were treated with IM, and bone marrow from mice in molecular remission was transplanted into tertiary recipients for therapy discontinuation studies. Tertiary recipients were injected with Alum or saline every 4 weeks. Repeated episodes of emergency granulopoiesis; **a** induced progressive granulocytosis in CML mice. Steady state granulopoiesis did not resume after the first Alum injection in mice with untreated CP-CML, or the second injection during an IM discontinuation attempt. Significance indicated by *, **, ***, or # (*p* < 0.001, *n* = 6); **b** impaired survival in mice with untreated CML, or during IM discontinuation; **c** induced rapid BC in mice with untreated CML compared with steady state; and **d** enhanced CP-relapse after IM discontinuation compared with steady state.

In control mice, Alum injection induced maximal peripheral granulocytosis and bone marrow myeloid progenitor expansion by 2 weeks, with steady state resumption by 4 (Supplemental Fig. 3) [21, 22]. This was repeated four times at 4 week intervals without death or debility in Wt mice [21, 22]. To study the impact emergency granulopoiesis on CML, secondary recipients of CP bone marrow were injected with Alum or saline every 4 weeks, starting 4 weeks post-transplantation. Similar to Icsbp/Irf8^−/−^ mice, we found exaggerated Alum-induced granulocytosis without steady state resumption between episodes (Fig. 5a). GFP+granulocytes were significantly increased, but GFP^−^ granulocytes were also dysregulated after Alum-injection in these mice. Survival in CP-CML mice significantly was shortened by Alum injection with 50% survival of 14 weeks compared with 26 weeks at steady state (Fig. 5b; fewer cells were injected compared with studies in Fig. 2).

Myeloid blasts emerged more rapidly in CML mice during repeated emergency granulopoiesis episodes compared with steady state (*p* < 0.001, *n* = 6). Mice at steady state first developed CP with half subsequently progressing to BC (Fig. 5c) [12]. However, all Alum injected mice developed BC (>15% circulating myeloid blasts), without prior CP (i.e., >10,000 GFP+circulating granulocytes). Blasts were not found in control, Wt mice under any condition, consistent with our prior work [21, 22].

### Emergency granulopoiesis enhances CP-relapse after therapy discontinuation

To investigate the impact of emergency granulopoiesis on relapse after therapy discontinuation, we performed similar studies with tertiary recipients from IM-treated mice. Mice were transplanted with bone marrow from secondary donors in IM-induced molecular remission (at 24 weeks), and injected with Alum or saline starting 4 weeks after transplantation. We found resolution of emergency granulopoiesis after the first Alum-injection in these mice, but GFP^+^ circulating granulocytes progressively during subsequent episodes (Fig. 5a). Alum-injection significantly decreased survival, with 50% survival of 14 weeks in this cohort compared with survival of all mice at 24+ weeks during steady state (*p* < 0.001, *n* = 8) (Fig. 5b).

Most recipients from IM-treated donors relapsed in CP after the third emergency granulopoiesis episode. One Alum treated mouse relapsed directly into lymphoid BC after the second injection, and another progressed from CP to myeloid BC at 16 weeks. None of the mice at steady state relapsed over the observation time period shown, but 20% relapsed in CP by 24+ weeks.

## Discussion

Our studies implicate Survivin in apoptosis resistance of CML-LSCs, persistence of these cells during TKI treatment, and relapse after therapy discontinuation. We found that Survivin inhibition (with Ym155) induced molecular remission as efficiently as IM in mice with CP-CML. Mice relapsed in stable CP during Ym155 treatment, unlike IM-treated mice which relapsed in CP, but with subsequent BC progression in 50%. The combination of IM + Ym155 prevented relapse during therapy or after discontinuation in CML mice. Conversely, adding a Xiap1 inhibitor (Embelin) to IM did not delay drug resistance or post therapy discontinuation relapse. It was of interest that the combination of IM + Embelin was associated with relapse in BC rather than CP; both during treatment and after therapy discontinuation.

These results suggest that an understanding of differentiation-stage-specific pathways, and validation in preclinical models, is essential for safe translation of molecular targeting to human clinical trials. Ym155 and Embelin are relatively specific for inhibition of Survivin or Xiap1, respectively. We previously demonstrated differentiation stage specific activity of these proteins with shRNA knockdown in Bcr-abl-transduced bone marrow cells [17]. In vivo confirmation with genetic models would verify this conclusion, but our studies identify the potential for treatment with Ym155 to permit more CML patients to qualify for therapy discontinuation or remain in TFR. This might be most relevant to selected patients with relapse after a first discontinuation attempt.

Survivin expression increased with CP or BC relapse during IM treatment. Both βcatenin and Gli1 paralleled survivin during this process, providing possible mechanisms. Increased Msi2 in BC induces differentiation block through Numb inhibition and Notch activation [26]. Decreased Msi2 in CP may represent an unsuccessful attempt to destabilize βcatenin by increasing Apc translation. Survivin-escape during IM treatment may involve multiple mechanisms; an area of interest for future investigations.

By transcriptome analysis, we found increased activity of innate immune response pathways in bone marrow progenitors from untreated CML mice compared with control. This was exaggerated by TKI-induced remission, although pathways involved in tyrosine kinase signaling and apoptosis were improved. The major transcriptome alteration upon adding Ym155 to IM was normalization of immune response pathways; suggesting their functional significance for LSC persistence and relapse. The addition of Ym155 also increased Irf8/Icsbp expression; an effect not observed with IM alone. Irf8/Icsbp functions as a CML suppressor by regulating genes that influence apoptosis, including the Fap1 and Gas2 genes [15, 16].

Studies in MDS and AML by other investigators identified mutations Traf6 or Uaf4 that constitutively activate inflammatory pathways [34, 35]. In contrast, we considered the role in leukemogenesis of a dysregulated physiologic response to infectious challenge. In prior studies, we found Irf8/Icsbp was necessary to terminate emergency granulopoiesis [21]. Decreased Irf8/Icsbp in CML suggested that the emergency granulopoiesis response might be sustained in this disease, and a failure of IM to correct this had implications for LSC-persistence and relapse.

Genotoxic stress is increased in emergency granulopoiesis by S phase shortening, accelerated differentiation, and generation of reactive oxygen species by accumulating bone marrow granulocytes [21, 22, 36]. Perhaps consistent with this, we found accelerated BC in mice with untreated CML during repeated emergency granulopoiesis episodes. Emergency granulopoiesis failed to terminate in these mice, suggesting a sustained response contributed to leukemogenesis. Accumulation of granulocytes in the bone marrow triggers termination of emergency granulopoiesis through unknown molecular mechanisms [23]. Enhanced apoptosis of CML granulocytes in Embelin-treated mice might impair this regulatory mechanism, worsening dysregulation of the innate immune response, and enhancing mutagenesis. This may explain relapse in BC rather than CP of CML mice treated with Embelin, with or without IM.

We found repeated episodes of emergency granulopoiesis during a TKI discontinuation attempt enhanced, and accelerated CP relapse in this murine model, but did not lead to BC. Most mice died from overwhelming granulocytosis prior to BC progression. We hypothesize IM-induced remission abolishes LSC subset(s) with enhanced susceptibility to mutagenesis and BC during the innate immune response. Studies to investigate this are ongoing in the laboratory.

Our findings suggest that impaired termination of emergency granulopoiesis increases the hazard of infectious challenge in CML. Decreased Irf8/Icsbp expression is also found in acute myeloid leukemia (AML) with t(8;21) and a subset of therapy-related AML [37, 38]. The homeodomain transcription factor HoxA9 impairs termination of emergency granulopoiesis by repressing Triad1, an E3 ubiquitin ligase and increased Hox expression is found in an adverse prognosis subset of AML [39, 40]. We hypothesize the inability to resume steady state granulopoiesis after an infectious challenge drives disease progression in subsets of myeloid leukemia. Further studies to determine if termination of emergency granulopoiesis is a physiologic equivalent of leukemia suppression are of interest.

## Supplementary information

Supplemental Figure 1

Supplemental Figure 2

Supplemental Figure 3
