# Supplementary figures and images for: Investigating the role of the innate immune response in relapse or blast crisis in chronic myeloid leukemia
Source: Leukemia. 2020 Feb 20;34(9):2364–74. doi: 10.1038/s41375-020-0771-7 (PMC7438233; doi:10.1038/s41375-020-0771-7)

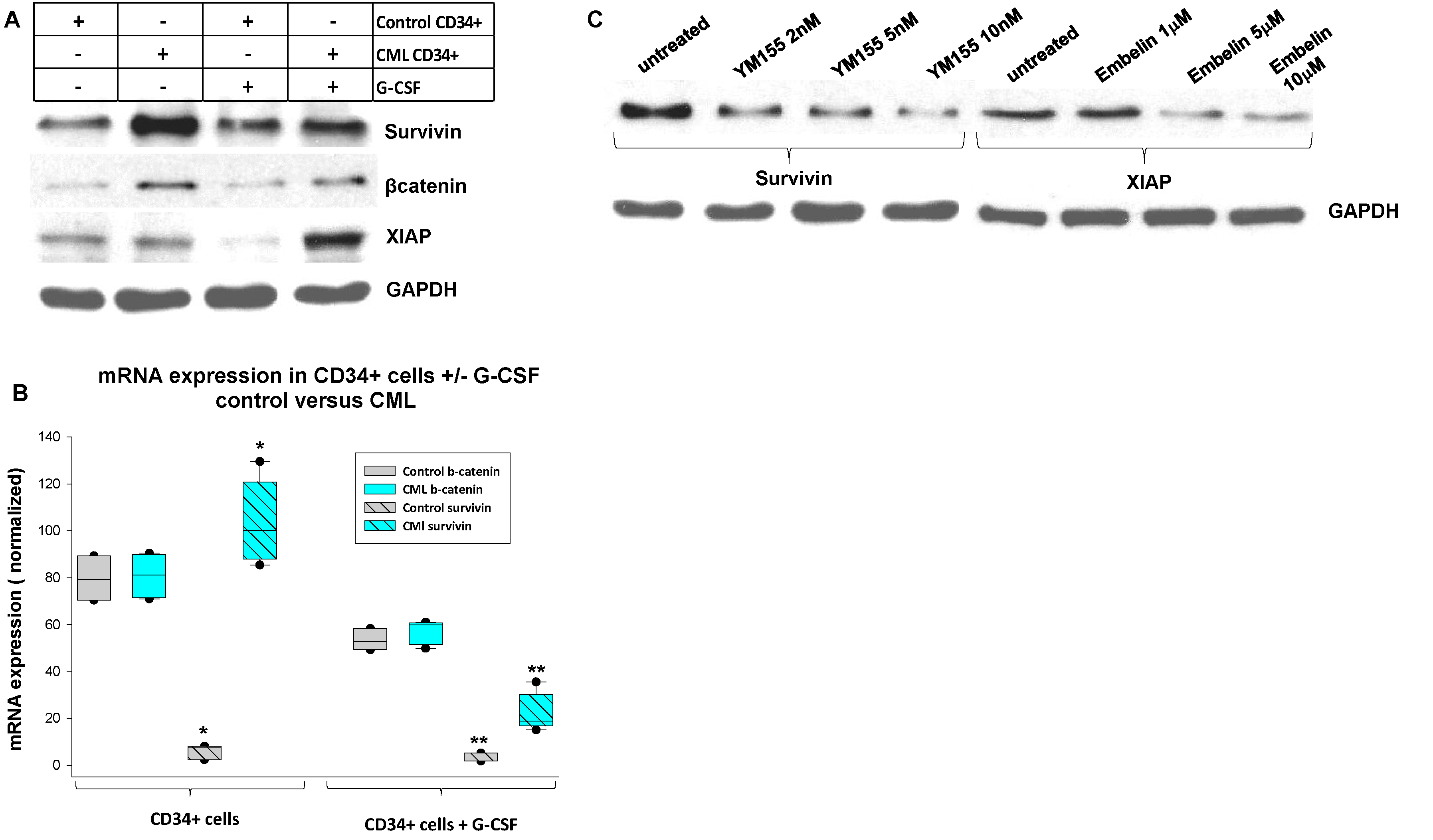

Supplement: Supplementary file 1 — Supplemental Figure 1 [file 41375_2020_771_MOESM1_ESM.tif]

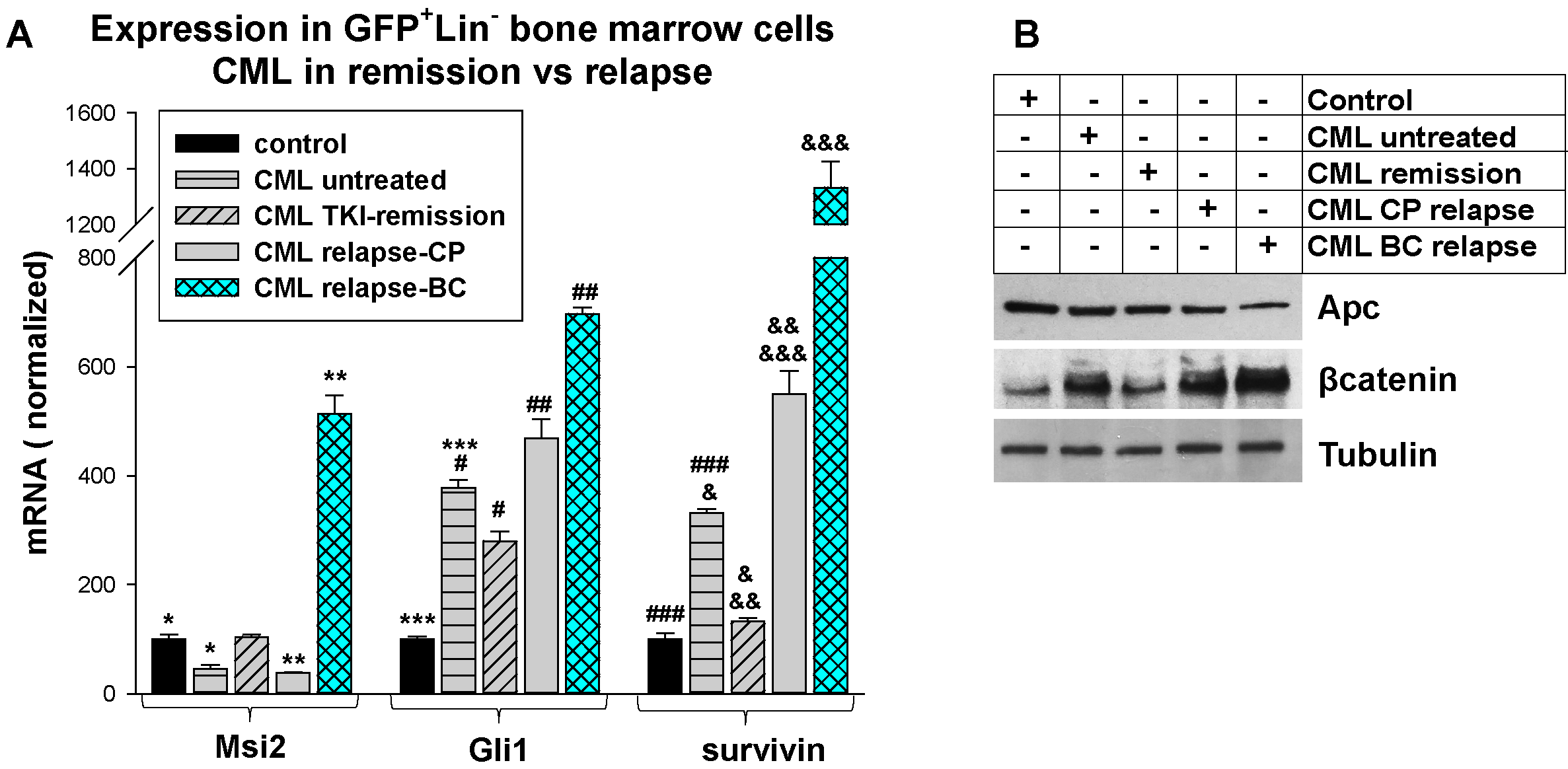

Supplement: Supplementary file 2 — Supplemental Figure 2 [file 41375_2020_771_MOESM2_ESM.tif]

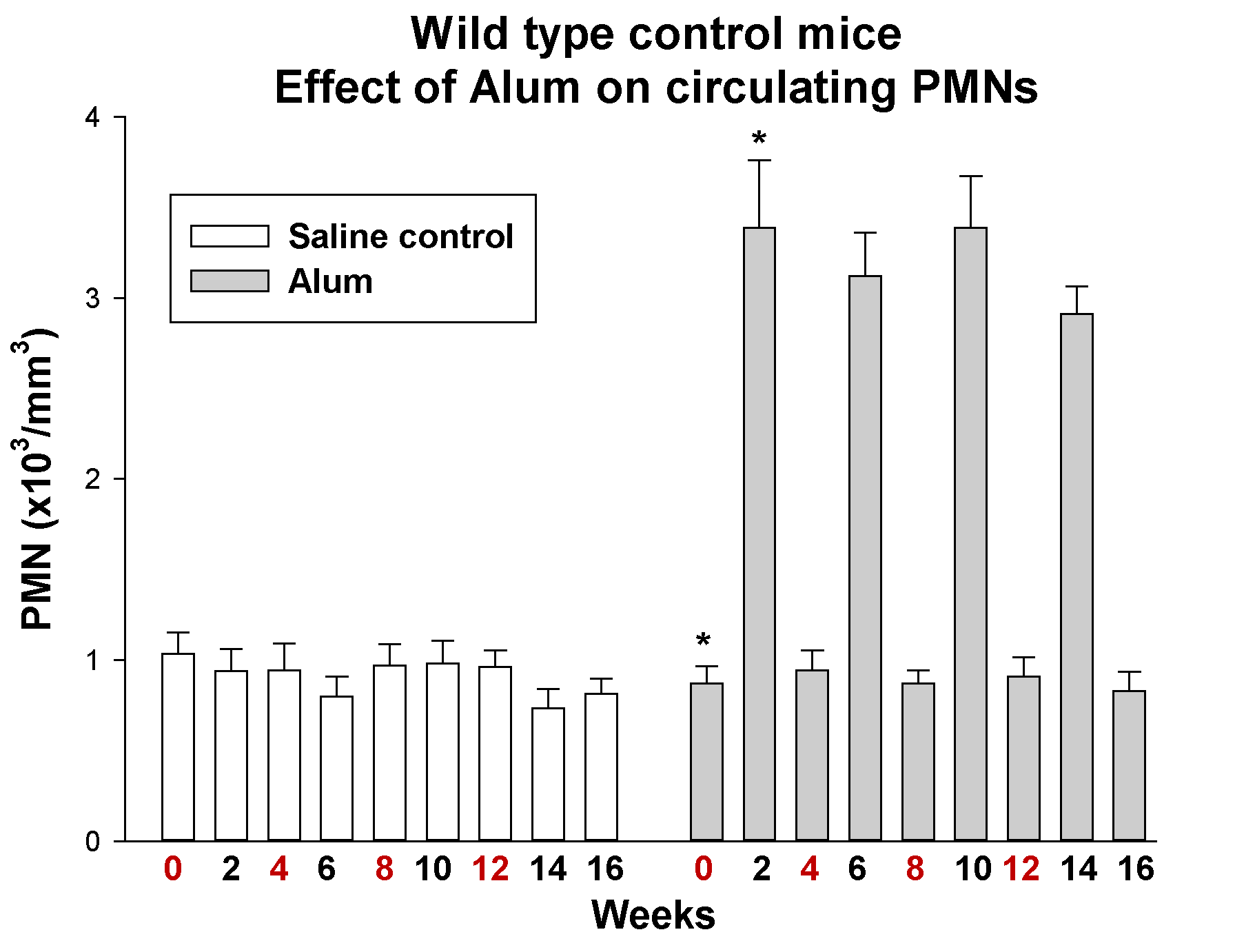

Supplement: Supplementary file 3 — Supplemental Figure 3 [file 41375_2020_771_MOESM3_ESM.tif]
